# Knowledge, Attitude, and Practice towards Evidence-Based Medicine among Northern Saudi Primary Care Physicians: A Cross-Sectional Study

**DOI:** 10.3390/healthcare10112285

**Published:** 2022-11-14

**Authors:** Bashayer Farhan ALruwaili, Ashokkumar Thirunavukkarasu, Aseel Awad Alsaidan, Aliyah Muteb AL-Ruwaili, Ruqayyah Batel Shati Alanazi, Amal Muhaysin B. Alruwaili, Abdullah Odhayb Alruwaili, Afrah Mohaimeed Altaymani

**Affiliations:** 1 Department of Community Medicine and Family Medicine, College of Medicine, Jouf University, Sakaka 72388, Aljouf, Saudi Arabia; 2 Department of Public Health, Ministry of Health, Sakaka 73244, Aljouf, Saudi Arabia; 3 Department of Family Medicine, Northern Borders General Health Affairs, Arar 73244, Northern Border Province, Saudi Arabia; 4 Department of Family Medicine, King Abdulaziz Specialist Hospital, Sakaka 72345, Aljouf, Saudi Arabia; 5 Medical Intern, College of Medicine, Jouf University, Sakaka 72388, Aljouf, Saudi Arabia

**Keywords:** evidence-based medicine, knowledge, attitude, Saudi, practice, barriers

## Abstract

The evidence-based practice of primary care physicians is essential because they are the first line of contact with the local community, and they cater to most of their communities’ health needs. In the current study, in which we used a cross-sectional survey in northern Saudi Arabia, we assessed primary care physicians’ knowledge, attitude, practice, and barriers regarding evidence-based medicine (EBM). Of the 300 physicians who participated, less than half had high knowledge (43.7%) and attitude (47.7%) toward EBM. The chi-square test revealed that the knowledge categories were significantly associated with the age group (*p* = 0.002) and EBM training received in the past five years (*p* < 0.001), and the attitude categories were significantly associated with nationality (*p* = 0.008). Of the respondents, 155 (51.7%) used EBM in their daily clinical practice. Through logistic regression analysis, we found that the identified predictors of including EBM in clinical practice were the 31–45-year-old age group (adjusted odds ratio (AOR) = 2.11, 95% confidence interval (CI) = 1.65–2.73) and EBM training received during last 5 years (AOR = 2.12, 95% CI = 1.35–2.94). We recommend enhancing primary care physicians’ knowledge of EBM and its importance in clinical practice through appropriate training programs. A multi-centric mixed-method survey is warranted in other provinces of the KSA to recognize region-specific training demand.

## 1. Introduction

Evidence-based medicine (EBM) is a method of systematically searching, critically appraising, and applying research findings and scientific evidence that help to deliver quality health care to patients [1]. The primary aim of EBM is to shift the patients’ care by physicians from studies with the lowest scientific evidence such as case reports, expert opinions, and academic authorities to using highly scientific evidence such as systematic reviews, meta-analyses, and clinical trials [1,2]. Evidence-based practice is a clinical practice that allows physicians to practice in their settings based on the best available scientific evidence [3]. Even though evidence-based practice started a few decades ago, policymakers have only emphasized it more recently during the COVID-19 pandemic, as many misconceptions and false information have circulated regarding vaccines, patient care, and COVID-19 patient management [4,5].

According to the World Health Organization (WHO), primary health care is essential, involves a holistic approach, and covers most people’s health needs. Primary care is not limited to disease management, as it also involves prevention, health promotion, rehabilitation, etc. [6]. In the Kingdom of Saudi Arabia (KSA), the Ministry of Health (MOH) is responsible for delivering public health to the community. Primary health care is delivered in the KSA through a chain of primary health centers (PHC), and this is the first level of contact for the public for all their health-related needs [7,8]. The evidence-based practice of primary care physicians is essential as they are the first line of contact with the local community and cater to most of their health needs [9]. Since primary healthcare is an important pillar for successful healthcare systems, delivering evidence-based healthcare at the level of PHCs would positively affect the community’s health.

Worldwide, 37.5% to 68.3% of primary care physicians have a positive attitude toward EBM [10,11,12]. Some studies have been conducted by KSA authors who assessed physicians’ and other healthcare providers’ awareness, attitude, and practice in different settings [13,14,15]. A recent survey conducted by Zanaridha MN et al. among Malaysian primary care physicians reported that less than one-third of them (32.9%) had substantial knowledge of EBM, 12% had a positive attitude toward EBM, and a very low proportion (0.4%) properly practiced EBM. They also revealed that EBM clinical practices are significantly related to ethnicity, work experience at PHCs, and access to EBM-related mobile applications [12]. A cross-sectional survey conducted by Al-Ansary LA et al. that assessed knowledge and attitude among primary healthcare physicians in the Riyadh region showed that their participants had low-level knowledge of journal extractions, publications, and relevant databases [16]. Moreover, the respondents had only limited interpretations of the technical words used in EBM. In their study, patient overload and time shortages were critical barriers to practicing EBM.

Due to the wide socio-cultural differences among healthcare workers and the population in the KSA, having region-specific data is essential. Based on our extensive literature review, we determined that this kind of study is the first to be conducted in the northern region of the KSA. An assessment of primary care physicians’ knowledge, attitudes, and practices of EBM might help policymakers and stakeholders formulate guidelines for region-specific training programs. This may also allow primary care physicians to provide services to people in a more scientific way. Hence, we conducted this study to assess the knowledge, attitudes, and practices of EBM among primary care physicians in northern KSA, and to determine factors associated with including EBM in their daily clinical practice. In the present survey, we also evaluated their perceived barriers to evidence-based practice.

## 2. Materials and Methods

### 2.1. Study Description

We conducted this PHC-based cross-sectional survey from April to August 2022 in four northern KSA provinces, namely, Hai’l, Aljawf, Tabuk, and the northern border. At the time of the study, these four regions had 317 PHCs and 1145 primary care physicians working.

### 2.2. Sampling Description

Using Cochran’s sample size equation (n = z^2^pq/e^2^), the present survey’s minimum number of required participants was computed [17]. In this equation, n = sample size, p = expected proportion at 50%, q = 1 − p, e = margin of error at 5%. We set the confidence interval (CI) at 95% and 80% of the study’s power. Applying the values mentioned earlier in the equation, with the known population of 1145, the necessary participants for the survey were measured as 288 and rounded to 300. The present research applied a simple random sampling method to select the study participants. In this technique, we entered all the physicians’ assigned numbers (1 to 1145) into the Statistical Package for the Social Sciences software (SPSS) with the principal investigator’s computer. Finally, we used SPSS to select 300 random physicians according to the assigned numbers. The data collectors invited the randomly selected primary care physicians to participate in the survey. In the event of a nonrespondent, the data collectors chose the next available and eligible participant from the same PHC.

### 2.3. Inclusion and Exclusion Criteria

The sampling frame included all primary care physicians of the 317 PHCs from all four provinces, and we excluded those who were reluctant to participate, primary care physicians who belonged to private sectors, other ministries, and PHCs affiliated with universities.

### 2.4. Ethical Consideration

Firstly, the present study protocol was approved by the Jouf University ethical committee (LCBE no: 1-08-43, dated 3 April 2022). After a briefing of the study, the primary care physicians gave informed consent to participate in the study. Finally, the survey form did not contain any identifying details of the respondents; thus, we maintained the anonymity of the participants.

### 2.5. Survey Questionnaire

The present study data were gathered using a pretested, standard, and validated data collection tool prepared by the research team based on available published papers [12,14,18,19]. A panel of family medicine, public health, and clinical research experts prepared the questionnaire, and we pretested the same questionnaire on 30 primary care physicians. All pilot study (pretest) physicians helped us to ensure that the data collection was clear pro forma. Moreover, the analysis of the pilot study did not reveal any missing data from the completed survey forms. Furthermore, we ensured the internal consistency of the questionnaire with Cronbach’s alpha values of 0.87, 0.79, and 0.93 for knowledge, attitude, and barriers, respectively. The study questionnaire had four parts: Firstly, it collected the participants’ sociodemographic characteristics and EBM training received by the participants during the past five years. Secondly, it inquired about practitioners’ knowledge and awareness of relevant clinical research databases and technical terms used in clinical research. The knowledge section consisted of 12 questions and was divided into two subsections. The first knowledge subsection consisted of 5 questions that inquired about primary care physicians’ knowledge of relevant EBM databases. In this subsection, participants responded on a 4-point Likert scale ranging from “not aware” (0 points) to “use it in clinical practice” (3 points). The second knowledge subsection consisted of 7 questions that inquired about technical terms used in EBM practice. For each technical term, physicians responded on a 4-point Likert scale ranging from “not aware, as it is not useful for clinical practice” (0 points) to “can interpret it and describe it to others” (3 points). The third part asked about their attitude toward EBM. This section contained five items, and the participants responded to each item according to the instructions, as follows: (A) attitude toward the ongoing promotion of EBM (0 = extremely unwelcoming, 100 = extremely welcoming); (B) perceived attitude of fellow physicians toward EBM (0 = extremely unwelcoming, 100 = extremely welcoming); (C) practicing EBM enhances patient management (0 = strongly disagree, 100 = strongly agree); (D) perceived use of EBM in day-to-day patient care (0 = no use, 100 = extremely useful); (E) approximate proportion of practice at PHCs that is evidence-based practice.

We computed and categorized the total knowledge and attitude scores into low (<60% of the total score), average (60 to 79% of the total score), and high (80% of the total score and above). Furthermore, we combined the low and average groups into a single group for further analysis according to Bloom’s classification. For the categorization in this study, we followed Bloom’s classification, which has been widely used by researchers [20,21]. The fourth part inquired about the usage and inclusion of EBM in their daily clinical practice. Finally, the primary care physicians answered “yes” or “no” regarding barriers to practicing EBM in their work settings.

### 2.6. Data Collection Procedure

The data collection team visited the workplaces of the selected primary care physicians. After explaining the current survey’s aim, we obtained informed consent. We requested the physicians who were willing to participate to complete an online survey form (Google form) on the research team’s mobile devices and tablets. We only permitted the principal investigator to access and download the Excel sheets, thus ensuring data security.

### 2.7. Statistical Analysis

The survey team used the Statistical Package for Social Sciences, version 21.0 (IBM Corp., Armonk, NY, USA) for analysis. We conducted descriptive statistical analysis to obtain frequency and measure proportions. A chi-square test was applied to identify the association between knowledge and attitude categories with background characteristics of the study population. Finally, logistic regression analysis was performed to identify the predictors of not including EBM in clinical practice. In this method, we analyzed each independent variable with the EBM practice. Next, we adjusted all the study variables: age, gender, physicians’ nationality, qualification, current position, PHC work experience, and EBM training in the past five years. We fixed an alpha (*p*) value of less than 0.05 as a significant value.

## 3. Results

Of the 300 study participants who responded, most of the physicians belonged to the 31–45 years old age group, (41.3%), male gender (63.0%), and Saudi nationalities (55.0%). Regarding professional qualifications, 43.3% were bachelor’s degree holders, 47.3% were residents, and 38.0% had work experience of fewer than five years. More than two-thirds (38.3%) of the participants had not received any training related to EBM in the past five years (Table 1).

The present survey participants’ knowledge and awareness of EBM-related databases are shown in Table 2. Of the 300 participants, about half (46.0%) read and use it for clinical practice, and 51.0% were unaware of the Saudi digital library and DARE.

Regarding the knowledge and awareness of some commonly used technical terms in EBM, 50.7% of participants responded that they could “interpret and describe the term ‘absolute risk’ to other primary care physicians”. Fewer than half of the participants reported that they could interpret and describe the remaining terms, which were “relative risk” (46.7%), “systematic review” (28.3%), “odds ratio” (36.0%), “meta-analysis” (38.0%), “clinical effectiveness” (30.7%), and “confidence interval” (31.3%) (Table 3).

The physicians’ attitudes toward EBM were as follows: (A) attitude toward the ongoing promotion of EBM (median: 75); (B) fellow physicians’ perceived attitude toward EBM (median: 62); (C) practicing EBM enhanced patient management (median: 70); (D) perceived use of EBM in day-to-day patient care (median: 65); (E) approximate proportion of practice at PHCs that is evidence-based practice (median: 60).

The chi-square analysis revealed that the knowledge categories were significantly associated with the age group (*p* = 0.002) and the EBM training received in the past five years (*p* < 0.001), and the attitude categories were significantly associated with nationality (*p* = 0.008), and the EBM training received in the past five years (*p* = 0.007) (Table 4).

Among the respondents, 155 (51.7%) used EBM in their daily clinical practice, 84 (28.0%) used it sometimes, and 61 (20.3%) never used EBM in their clinical practice. We analyzed the predictors for including EBM in daily clinical practice by binomial logistic regression analysis, as depicted in Table 5 and Table 6. First, we carried out a univariate analysis (Table 5) followed by a multivariate analysis (binomial logistic regression analysis: enter method). The significant predictors that we identified through the multivariate analysis (after adjusting for potential confounders) were the 31–45-year-old age group (adjusted odds ratio (AOR) = 2.11, 95% confidence interval (CI) = 1.65–2.73), age group ≤ 30 years (AOR = 1.29, 95% CI = 1.08–1.52), having work experience of more than 10 years (AOR = 0.57, 95% CI = 0.39–0.71), and having received EBM training in the last 5 years (AOR = 2.12, 95% CI = 1.35–2.94).

The most common barriers perceived by the respondents were “patients’ values, concerns, and expectations (56.3%)”, followed by “lack of adequate training in EBM (44.0%)”, “lack of clarity about roles and practice (43.3%)”, and “workplace culture (40.3%)” (Table 7).

## 4. Discussion

Evidence-based practice has become a crucial component of general practice and primary healthcare delivery, especially with the public’s expanding access to health-related information and demand for accountability. The present survey assessed primary care physicians’ knowledge, attitude, and practice towards EBM and the barriers to including EBM in their clinical practice.

Healthcare delivery, including primary care at PHCs, is driven by knowledge among healthcare providers [19,22]. Regarding the knowledge and awareness of relevant EBM databases (modified for KSA settings) in the present survey, a low proportion of the participants (PubMed—46%, DARE—23.3%, and Saudi Digital Library—16.7%) were able to read and use it for their clinical practice. Similarly, a questionnaire-based study conducted by McColl et al. also stated that general practitioners had low awareness and access to the commonly used EBM databases [18]. Our findings are consistent with a recent cross-sectional survey conducted in Croatia by Nejašmić D et al. They explored that less than half of the family physicians considered the major EBM database (Medline—49.1% and Cochrane library—47.1%) useful for patient care [23]. Interestingly Unadkat, M.B et al. 2021 reported a good understanding of EBM among their study respondents [24]. These striking dissimilarities were perhaps the result of the diversity of the responding doctors. The current research participants comprised all levels of primary care physicians, but Unadkat, M.B et al. included only residents in their survey.

Upon responding to the awareness-related questions of EBM technical terms, about half of the participants noted that they could interpret and explain “relative risk” (46.7%) and “absolute risk” (50.7%) to other doctors. However, less than one-third of them could interpret and explain other terms, such as “systematic review” (28.3%), “clinical effectiveness” (30.7%), and “confidence interval” (31.3%). In a survey conducted among physicians in the Qassim region of the KSA, Alshehri et al. noted that a lower proportion of physicians were aware of the terms “relative risk” (40.3%), “absolute risk” (40.6%), and “confidence interval” (17.3%) [13]. In contrast, a higher proportion of respondents were found to be aware of EBM technical terms in a study conducted in Kuwait by Qadhi et al. [19]. The differences in the studies mentioned above might be due to the diversity of the included participants. Our respondents were primary care doctors from different PHCs, and the mentioned studies had physicians from other settings.

We found that nearly 44% of the participants had high knowledge of EBM. In a recent survey that was conducted in Malaysia, less than one-third of the study’s primary care physicians were found to have substantial knowledge of EBM [12]. We found that primary care physicians’ knowledge of EBM was significantly associated with age group (*p* = 0.002) and EBM training received in the past five years (*p* < 0.001). Similarly, EBM-related knowledge was significantly higher among doctors who received formal training for EBM [19]. A study conducted in Kenya by Unadkat et al. reported that age is not a significant factor related to EBM knowledge [24]. The possible dissimilarities between the current study and this study could be the inclusion criteria of physicians. Our study included only primary care physicians.

A positive attitude is a fundamental requirement for healthcare workers, and previous studies have proven that a positive attitude among primary care professionals is associated with better healthcare delivery [25,26]. We set cut-off values according to Bloom’s criteria, which was applied in several studies in the KSA [27,28]. This PHC-based study explored that primary care physicians had suboptimal scores in all five attitude aspects and the attitude categories were significantly associated with nationality (*p* = 0.008) and EBM training received in the last 5 years (*p* = 0.007). In contrast to the present study, Alshehri et al. and Abdel-Kareem et al. participants had a higher proportion of positive attitudes towards EBM [13,29]. However, their participants belonged to secondary and tertiary care centers, but the current study respondents were from primary health centers. Identical to our findings, Bin Briek et al. also found suboptimal attitudes among the physicians who participated in their study [30]. A study conducted by Hong J in China reported a positive attitude among the clinical physicians working in the hospitals [31]. These wide variations in the findings from different parts of the world could be attributed to cultural factors, work settings, and EBM training requirements by the concerned health authorities.

The present study explored that more than half (51.7%) of primary care physicians were including EBM in their daily clinical practice, and it was significantly associated with age group (*p* = 0.028), PHC work experience (*p* = 0.007), and EBM training during last five years (*p* = 0.001). A survey conducted in China in 2019 by Hong J et al. showed a higher rate of physicians often practicing EBM [31]. They also reported that EBM practice was significantly associated with the workload and personal interests of the physicians. Another study executed in Malaysia by Ahmad G et al. contradicts the findings of the present survey. In their research, the predictors for EBM practices were gender and ethnicity [32]. Similar to this research, Qadhi et al. showed that 52.1% of their participants feel that their patients’ care is evidence-based [19]. A study by Zanaridah MN et al. also found that work experience duration is an essential predictor of EBM practice among primary care physicians [12].

The barriers to EBM practice can be related to personal, social, and work-related aspects. Overcoming the obstacles to EBM practice is critical for successfully implementing evidence-based practice in primary health care [33,34]. The findings of the present study suggest that age is one of the strongest predictors of evidence-based practice. The odds of including EBM in daily clinical practice are higher in younger age groups. In an assessment of variations in EBM practice through a qualitative study among primary care physicians, Hisham et al. found that physicians had different opinions regarding the best way to care for their patients. Senior physicians believed that patient care based on their clinical experience was more efficient than practicing EBM [35]. The findings of our study are supported by a systematic review performed by Choudhry et al., who concluded that senior physicians were less likely to practice the latest evidence-based care, leading to decreases in the quality of patient care [36]. The commonest barriers perceived by the present study’s respondents were “patients’ values, concerns, and expectations”, “lack of adequate training in EBM”, “lack of clarity about roles and practice”, and “workplace culture”. Several studies globally carried out have found different barriers [33,37]. A study by Khammarnia et al. in Iran stated that lack of human resources and time are their significant barriers [37]. Another study in the KSA revealed that inadequate facilities and workplace cooperation were substantial barriers to EBM practice [38]. A study conducted in Egypt stated that colleagues’ attitudes (workplace culture), workload, and lack of time were the major barriers for their study participants [30]. Another recent survey by Unadkat MB et al. reported a similar finding to the present research. Their survey’s most common barriers were lack of EBM knowledge and training and patient-related factors [24]. In another study, Li et al. reported that focusing on the workplace and organizational context is essential for the implementation of evidence-based practice in an organization, including primary health centers. Six contextual workplace features, workplace culture, encouragement from leaders, colleague networking, resources, assessments, and monitoring, were reported to influence the implementation of EBM [38].

Although this survey was conducted with a proper methodology using a pretested and validated tool, the readers of the research must consider some limitations of the survey. Firstly, we followed a cross-sectional study protocol, and thus, the limitations of the cross-sectional studies should be considered. Next, even though our study included primary care physicians of all nationalities, the sociocultural characteristics of the population of northern Saudi could be different from other parts of the KSA. Therefore, we cannot generalize our survey’s findings to other regions of KSA and other Middle East countries. The data of the present survey were self-reported. Hence, bias related to self-reported surveys, such as subjectivity and exaggerated reporting and recall, cannot be ignored. Furthermore, we used closed-ended listed barriers to assess the barriers to EBM practice. Hence, the present study could identify the prevalence of some of the barriers to EBM practice. Hence, we recommend using focused group discussions and mixed-method surveys in future research.

## 5. Conclusions

From this PHC-based survey, we found that more than half of the physicians had either low or medium knowledge and attitude toward EBM. Possessing EBM-related knowledge was significantly associated with the age group, the EBM training received in the past five years, and the attitude categories were significantly associated with nationality. This survey found that only about half of primary care physicians include EBM in their daily clinical practice, which was significantly associated with age, PHC work experience, and lack of EBM training in the past five years. The perceived barriers to EBM practice were patients related and workplace culture. Hence, we recommend enhancing primary care physicians’ knowledge of EBM and its importance in clinical practice through appropriate training programs. Furthermore, decision-makers must incorporate strategies to overcome the barriers to EBM practice. Finally, a multi-centric mixed-method survey should be conducted in other provinces of the KSA to recognize region-specific training demand.

## Figures and Tables

**Table 1:** Background details of study participants (n = 300).

Background Details of Physicians	Frequency	Percentage
Age in years (mean ± SD)	36.2 ± 6.7	
≤30	90	30
31–45	124	41.3
>45	86	28.7
Gender		
Male	189	63
Female	111	37
Physicians’ nationality		
Saudi	165	55
Non-Saudi	135	45
Highest qualification		
Bachelor (MBBS/MBBCh/Equivalent)	130	43.3
Masters (MD/MS)	102	34
Saudi Board and Fellowship	68	22.7
Current position		
Resident	142	47.3
Specialist	95	31.7
Consultant	63	21
PHC work experience		
Up to 5 years	114	38
6 to 10 years	96	32
More than 10 years	90	30
EBM training in the last 5 years		
No	115	38.3
Yes	185	61.7

**Table 2:** Knowledge and awareness regarding relevant EBM databases (n = 300).

Database	Not Aware No (%)	Aware but Never Used No (%)	Read but Never Used It No (%)	Read and Use It for Clinical Practice No (%)
Journal of Health Specialties by Saudi Commission	52 (17.3)	88 (29.3)	91 (30.3)	69 (23.0)
PubMed (Medline)	44 (14.7)	71 (23.7)	47 (15.7)	138 (46.0)
Cochrane Library	132 (44.0)	85 (28.3)	60 (20.0)	23 (7.7)
Database of Abstracts of Reviews of Effects (DARE)	153 (51.0)	66 (22.0)	10 (3.3)	71 (23.7)
Saudi Digital Library	153 (51.0)	60 (20.0)	38 (12.7)	49 (16.3)

**Table 3:** Knowledge and awareness regarding technical terms used in EBM (n = 300).

Term	It May Not Be Useful for Me in Clinical Practice No (%)	Do Not Understand but Have the Intention to Learn No (%)	Some Interpretation No (%)	Interpret and Could Describe to Others No (%)
Relative risk	33 (11.0)	34 (11.3)	93 (31.0)	140 (46.7)
Absolute risk	17 (5.7)	27 (9.0)	104 (34.7)	152 (50.7)
Systematic review	27 (9.0)	33 (11.0)	155 (51.7)	85 (28.3)
Odds ratio	36 (12.0)	48 (16.0)	108 (36.0)	108 (36.0)
Meta-analysis	50 (16.7)	40 (13.3)	96 (32.0)	114 (38.0)
Clinical effectiveness	26 (8.7)	64 (21.3)	118 (39.3)	92 (30.7)
Confidence interval	35 (11.7)	78 (26.0)	93 (31.0)	94 (31.3)

**Table 4:** Association between knowledge and attitude with the background characteristics of the study population (n = 300).

		Knowledge			Attitude		
	Total (n = 300)	Low/Average (n = 169)	High (n = 131)	*p*-Value	Low/Average (n = 157)	High (n = 143)	*p*-Value
Age group							
≤30	90	37	53	0.002 *	44	46	0.202
31–45	124	80	44		61	63	
>45	86	52	34		52	34	
Gender							
Male	191	103	88	0.266	102	89	0.623
Female	109	66	43		55	54	
Physicians’ nationality							
Saudi	165	94	71	0.805	75	90	0.008
Non-Saudi	135	75	60		82	53	
Highest qualification							
Bachelor (MBBS/MBBCh/Equivalent)	130	71	59	0.511	66	64	0.406
Masters (MD/MS)	102	62	40		58	44	
Saudi Board and Fellowship	68	36	32		33	35	
Current position							
Resident	142	78	64	0.503	70	72	0.461
Specialist	95	58	37		50	45	
Consultant	63	33	30		37	26	
PHC work experience							
Up to 5 years	114	63	51	0.955	65	49	0.403
6 to 10 years	96	55	41		49	47	
More than 10 years	90	51	39		43	47	
EBM training in the last 5 years							
No	115	45	70	<0.001 *	49	66	0.007 *
Yes	185	124	61		108	77	

* Significant association (*p* < 0.05) obtained through chi-square test.

**Table 5:** Binomial logistic regression analysis between background characteristics and EBM practice (unadjusted).

Background Characteristics	Total	EBM in Clinical Practice		Unadjusted/Univariate		
		Never/ Sometimes (n = 145)	Daily (n = 155)	Odds Ratio (OR)/Beta Coefficient (β)	95% Confidence Interval (CI) of OR/exp (β)	*p*-Value
Age in years						
>45	86	32	54	Reference (ref)		
31–45	124	74	50	2.5	1.42–4.40	0.001 *
≤30	90	39	51	1.19	0.70–1.36	0.441
Gender						
Male	191	98	93	Ref		
Female	109	47	62	0.72	0.45–1.16	0.188
Physicians’ nationality						
Saudi	165	67	68	Ref		
Non-Saudi	135	78	87	0.91	0.58–1.43	0.728
Highest qualification						
Bachelor	130	58	72	Ref		
Masters (MD/MS)	102	53	49	1.34	0.79–2.26	0.291
Saudi Board and Fellowship	68	34	34	1.24	0.69–2.23	0.549
Current position						
Resident	142	64	78	Ref		
Specialist	95	48	47	1.24	0.74–2.09	0.428
Consultant	63	33	30	1.34	0.74–2.43	0.365
PHC work experience						
Up to 5 years	114	45	69	Ref		
6 to 10 years	96	43	53	1.24	0.72–2.16	0.483
More than 10 years	90	57	33	0.78	0.61–0.93	0.001 *
EBM training in last 5 years						
No	115	71	44	Ref		
Yes	185	74	111	2.82	1.91–3.71	0.002 *

* Significant *p*-value at 0.05.

**Table 6:** Predictors for including EBM in daily clinical practice analyzed by binomial logistic regression analysis (n = 300).

Background Characteristics	Total	EBM in Clinical Practice				
		Never/ Sometimes (n = 145)	Daily (n = 155)	Adjusted OR/β *	95% CI of OR/β	*p*-Value
Age in years						
>45	86	32	54	Ref		
31–45	124	74	50	2.11	1.65–2.73	0.001 **
≤30	90	39	51	1.29	1.08–1.52	0.028 **
Gender						
Male	189	95	94	Ref		
Female	111	50	61	0.9	0.53–1.52	0.697
Physicians’ nationality						
Saudi	165	67	68	Ref		
Non-Saudi	135	78	87	1.02	0.63–1.67	0.535
Highest qualification						
Bachelor	130	58	72	Ref		
Masters (MD/MS)	102	53	49	1.15	0.53–2.52	0.731
Saudi Board and Fellowship	68	34	34	1.03	0.47–2.18	0.447
Current position						
Resident	142	64	78	Ref		
Specialist	95	48	47	1.12	0.57–2.18	0.746
Consultant	63	33	30	1.05	0.51–1.97	0.889
PHC Work experience						
Up to 5 years	114	45	69	Ref		
6 to 10 years	96	43	53	0.84	0.43–1.65	0.612
More than 10 years	90	57	33	0.57	0.39–0.71	0.007 **
EBM Training in the last 5 years						
No	115	71	44	Ref		
Yes	185	74	111	2.12	1.35–2.94	0.001 **

* Adjusted variables: age, gender, physicians’ nationality, qualification, current position, PHC work experience, EBM training in past 5 years; ** significant value at 0.05.

**Table 7:** Barriers to evidence-based practice (n = 300).

Barriers	Presence of Barriers	
	Frequency	Proportion
Lack of clarity about roles and practice	130	43.3
Lack of motivation	113	37.7
Workplace culture	121	40.3
Constraints of accessing EBM resource	88	29.3
Patients’ values, concerns and expectations	169	56.3
Lack of adequate training on EBM	132	44.0
Lack of time/Time mismanagement	95	31.7

## Data Availability

The data used to analyze this survey will be provided by the corresponding author upon request.
